# Discovery of Small-Molecule Inhibitors Against Norovirus 3CL^pro^ Using Structure-Based Virtual Screening and FlipGFP Assay

**DOI:** 10.3390/v17060814

**Published:** 2025-06-04

**Authors:** Hao Shen, Shiqi Liu, Limin Shang, Yuchen Liu, Yijin Sha, Dingwei Lei, Yuehui Zhang, Chaozhi Jin, Shanshan Wu, Mingyang Zhang, Han Wen, Chenxi Jia, Jian Wang

**Affiliations:** 1 School of Basic Medical Sciences, Anhui Medical University, Hefei 230032, China; exaid03052025@163.com (H.S.); lsq1110200@163.com (S.L.); 18168971225@163.com (Y.S.); 2 State Key Laboratory of Medical Proteomics, Beijing Proteome Research Center, National Center for Protein Sciences (Beijing), Beijing Institute of Lifeomics, Beijing 102206, China; data.cool@163.com (L.S.); liuyuchen@ncpsb.org.cn (Y.L.); qyhhzyh@163.com (Y.Z.); jinx-1@163.com (C.J.); wushanshan870227@163.com (S.W.); mingyangzhang001@163.com (M.Z.); 3 School of Pharmaceutical Sciences, Peking University, Beijing 100091, China; dwlei@stu.pku.edu.cn; 4 AI for Science Institute, Beijing 100085, China; wenh@aisi.ac.cn

**Keywords:** norovirus, 3C-like protease (3CL^pro^), virtual screening, FlipGFP assay, antiviral drug discovery, molecular docking and dynamics

## Abstract

Norovirus, a major cause of acute gastroenteritis, possesses a single-stranded positive-sense RNA genome. The viral 3C-like cysteine protease (3CL^pro^) plays a critical role in processing the viral polyprotein into mature non-structural proteins, a step essential for viral replication. Targeting 3CL^pro^ has emerged as a promising strategy for developing small-molecule inhibitors against Norovirus. In this study, we employed a combination of virtual screening and the FlipGFP assay to identify potential inhibitors targeting the 3CL^pro^ of Norovirus genotype GII.4. A library of approximately 58,800 compounds was screened using AutoDock Vina tool, yielding 20 candidate compounds based on their Max Affinity scores. These compounds were subsequently evaluated using a cell-based FlipGFP assay. Among them, eight compounds demonstrated significant inhibitory activity against 3CL^pro^, with Gedatolisib showing the most potent effect (IC_50_ = 0.06 ± 0.01 μM). Molecular docking and molecular dynamics simulations were conducted to explore the binding mechanisms and structural stability of the inhibitor–3CL^pro^ complexes. Our findings provide valuable insights into the development of antiviral drugs targeting Norovirus 3CL^pro^, offering potential therapeutic strategies to combat Norovirus infections.

## 1. Introduction

Human Norovirus (*Caliciviridae* family and *Norovirus* genus) is the predominant cause of acute gastroenteritis globally [1]. It is estimated to contribute to approximately 20% of all diarrheal cases annually, with severe infections leading to significant morbidity and mortality, particularly among children, the elderly, and immunocompromised individuals. Annually, Norovirus is responsible for nearly 200,000 deaths, with the majority occurring in children from low-income countries [2,3]. The highly contagious nature of Norovirus facilitates rapid transmission in densely populated settings such as schools and hospitals, underscoring the urgent need for effective prevention and treatment strategies.

Norovirus exhibits extensive genetic diversity, with seven genogroups (GI–GVII) identified to date, among which genogroup II (GII) is the most prevalent in human infections [4]. Within this genogroup, GII.4 strains have been responsible for the majority of global Norovirus outbreaks [5,6]. The periodic emergence of novel GII.4 variants, driven by antigenic drift, has been linked to global pandemics occurring approximately every decade [7]. This genetic variability poses a significant challenge for vaccine development and highlights the importance of targeting conserved viral elements, such as the 3CL^pro^, in antiviral drug design.

Structurally, Norovirus is a non-enveloped, single-stranded, positive-sense RNA virus capable of infecting a range of mammalian species. The human Norovirus genome, approximately 7.5 kb in length, comprises three open reading frames (ORF1–3). ORF1 encodes a polyprotein that is proteolytically cleaved by 3CL^pro^ to generate six essential nonstructural proteins: NS1/2, NS3 (helicase), NS4 (3A-like protein), NS5 (VPg), NS6 (3CL^pro^), and NS7 (RNA-dependent RNA polymerase, RdRp) [4,8]. The critical role of 3CL^pro^ in polyprotein processing makes it an attractive target for antiviral drug development [9]. Structurally, Norovirus 3CL^pro^ is a cysteine protease with chymotrypsin-like folds, consisting of an N-terminal antiparallel β-sheet domain and a C-terminal β-barrel domain. The catalytic triad (His30/Cys139/Glu54) resides within the cleft between these domains [8,10,11]. Previous efforts have identified protease inhibitors targeting 3CL^pro^, including the dipeptidyl compound GC376 [12,13,14]. The amino acid identities of different Norovirus 3CL^pro^ were found to be approximately 90% within the genogroup and 50–70% among different genogroups, which necessitates the development of next-generation inhibitors with broad-spectrum activity [12].

Computational approaches have become integral to modern drug discovery, enabling the efficient identification and optimization of lead compounds [15]. Virtual screening techniques, particularly ligand-based and structure-based methods, are widely employed to identify promising drug candidates [16]. Molecular docking, a cornerstone of structure-based drug design, involves predicting the binding conformation and orientation of small molecules within a target protein’s active site [17]. This method relies on scoring functions to evaluate binding affinity and identify low-energy conformations, facilitating the selection of candidate compounds for experimental validation [18].

In this study, we employed a combination of virtual screening and the FlipGFP-based assay to screen for potential inhibitors of Norovirus 3CL^pro^, using GC376 as a reference compound. Candidate inhibitors were first selected through structure-based virtual screening from a curated compound library. Their inhibitory activity was then validated in a cellular system using the FlipGFP assay. Our findings demonstrate that the virtual screening and FlipGFP methods enable the efficient identification of 3CL^pro^ inhibitors, providing a valuable platform for future antiviral drug development against Norovirus.

## 2. Materials and Methods

### 2.1. Plasmid Construction

The DNA sequence encoding Norovirus 3CL^pro^ (GenBank ID: PQ213805.1) was synthesized by General Biol (Chuzhou, China) and subsequently cloned into the pLVX-IRES-Puro vector. Constructs were generated using standard molecular biology techniques, including restriction enzyme digestion (New England BioLabs, Ipswich, MA, USA) and ligation with T4 DNA ligase (New England BioLabs, Ipswich, MA, USA). PCR amplification was performed using KOD One™ PCR Master Mix (TOYOBO, Osaka, Japan), following the manufacturer’s protocols. The resulting plasmids were transformed into Trelief^®^ Chemically Competent Cells (TSC-C01, Tsingke, Beijing, China) for amplification. All constructs were verified by Sanger sequencing, which was conducted by an external vendor (SinoGenoMax, Beijing, China).

### 2.2. Cell Culture and Transfection

Human embryonic kidney cells (HEK293T, ATCC: CRL-11268) were maintained in Dulbecco’s Modified Eagle’s Medium (DMEM; Gibco, Billings, MT, USA) supplemented with 10% (*v*/*v*) fetal bovine serum (FBS; ExCell, Buellton, CA, USA) at 37 °C in a humidified 5% CO_2_ atmosphere. Cell density and viability were assessed using an Automated Cell Counter (TC20™, Bio-Rad, Hercules, CA, USA). For transfection experiments, HEK293T cells were seeded into 24-well plates at a density of 1.5 × 10^5^ cells per well and allowed to adhere for 24 h. Transfection mixtures were prepared by combining 250 ng of fluorescent protein plasmid and 250 ng of protease plasmid in 50 μL of serum-free DMEM, followed by the addition of 1500 ng polyethyleneimine (PEI; Polysciences, Niles, IL, USA). After incubating the mixture at room temperature for 20 min, it was added dropwise to the cells. Six hours post-transfection, the medium was replaced with fresh DMEM containing 10% FBS and small-molecule compounds at varying concentrations. Fluorescent protein expression was analyzed 24 h after transfection.

### 2.3. Fluorescence Imaging and Fluorescence Intensity Measurement

At 24 h post-transfection, the fluorescence intensity of transfected cells in 24-well plates was quantified using a high-content screening system (PE-OPERETTA, PerkinElmer, Waltham, MA, USA). Green fluorescence was captured with an exposure time of 200 ms. Data were exported using Harmony 4.8 software and subsequently analyzed using GraphPad Prism 8.0 software (La Jolla, CA, USA) to determine fluorescence intensity and generate dose–response curves.

### 2.4. FlipGFP Assay

The protease inhibitor GC376, previously reported for its synthesis and activity against Norovirus 3CL^pro^ in both enzyme and cell-based assays [13], was utilized as a positive control in this study. A stock solution of GC376 (10 mM) was prepared in DMSO and diluted in DMEM to ensure a final DMSO concentration of 0.1% (vol/vol) in all assays. For the assay, 3CL^pro^ was incubated with FlipGFP in 50 µL of DMEM for 30 min. Transfection complexes were prepared using polyethyleneimine (PEI; Polysciences, PA, USA) at a concentration of 1 mg/mL and added to HEK293T cells seeded in 24-well plates. After 6 h of incubation at 37 °C, GC376 was added at concentrations ranging from 0.01 to 100 µM in 500 µL of DMEM. Fluorescence signals were measured using a high-content screening system with excitation and emission wavelengths of 490 nm and 520 nm, respectively. Mean fluorescence intensity (MFI) was calculated for each well, and dose-dependent inhibition curves were generated using GraphPad Prism 8.0 software (La Jolla, CA) with a variable slope (three-parameter) model to determine IC_50_ values. This protocol was consistently applied for the validation of all candidate compounds unless otherwise specified.

### 2.5. Docking Library Preparation and Molecular Docking

A compound library was curated by downloading and processing structures using OpenBabel (version 3.1.1), ensuring the removal of duplicates to yield a unique set of 58,836 compounds. This library was employed for molecular docking-based virtual screening against Norovirus 3CL^pro^ (PDB: 8U1V), which was modeled using AutoDock Vina (version 1.2.5). We selected the ligand-free crystal structure of Norovirus 3CL^pro^ (PDB ID: 8U1V) for molecular docking to preserve the native conformation of the binding site and avoid potential bias introduced by pre-bound ligands. Docking was performed using AutoDock Vina with the following parameters: (i) a docking grid centered at coordinates (x = −2.62, y = 24.955, z = 4.957) and a grid box size of 88.94 Å × 83.847 Å × 84.821 Å; (ii) an energy range of 5, a maximum of 9 docking modes, exhaustiveness values of 1, 8, and 16 across three rounds of screening, and a random seed of 2. From the initial library, 303 compounds were identified as potential hits based on docking scores. Compounds with Max Affinity scores below −10 were prioritized, and commercially available molecules were procured for further validation using the FlipGFP assay. The docking scores of the final eight hit compounds are calculated.

### 2.6. Molecular Dynamics Simulations

The molecular dynamics (MD) simulations were conducted using GROMACS 2022 and the total time for MD simulations was 100 ns. The PDBQT files of the eight hit compounds were converted to SDF format using OpenBabel (version 3.1.1), with hydrogen atoms added at pH 7. Target protein structures were repaired using PDBFixer to generate PDB files suitable for MD simulations. The GRAFF2 force field was applied to small-molecule ligands, while the AMBER99SB force field was used for proteins. A cubic simulation box of 10 × 10 × 10 nm^3^ was constructed, solvated with water molecules, and neutralized with chloride (Cl^−^) and sodium (Na^+^) ions at a concentration of 0.15 M. The simulation protocol included energy minimization, NVT (constant temperature) equilibration, NPT (constant pressure) equilibration, and a production run of 50,000,000 steps with a 2 fs time step. Simulations were performed at 290 K and 1.0 bar, with coordinates saved every 100 ps. The LINCS algorithm constrained hydrogen bonds, and the Verlet cut-off scheme was used for neighbor searching. Electrostatic interactions were handled using the particle mesh Ewald method, while temperature and pressure were controlled using the modified Berendsen thermostat and Parrinello–Rahman method, respectively. Three-dimensional periodic boundary conditions were applied, and initial velocities were not generated to ensure system stability. A trajectory analysis, including root mean square deviation (RMSD), root mean square fluctuation (RMSF), radius of gyration (Rg), and solvent-accessible surface area (SASA), was performed using built-in tools in the GROMACS package [19,20].

### 2.7. Western Blot

Whole cells were lysed in NETN buffer (20 mM Tris-HCl pH 7.6, 150 mM NaCl, 1 mM EDTA, 0.5% NP-40). Equal amounts of protein from each sample were resolved by SDS-PAGE and transferred to nitrocellulose (NC) membranes (PALL). The membranes were blocked with 5% skim milk in TBST (20 mM Tris-HCl pH 7.6, 150 mM NaCl, 0.05% Tween-20), followed by overnight incubation at 4 °C with primary antibodies (Flag-HRP, Myc-HRP, or GAPDH-HRP). After washing three times with TBST, target proteins were detected using ECL Western Blotting Substrate (Thermo, Waltham, MA, USA) and visualized with a ChemiDoc Imaging System (Bio-Rad).

## 3. Results

### 3.1. Development of a Flipgfp Assay for Detecting Norovirus 3CL^pro^ Inhibitors

**Figure 1:**
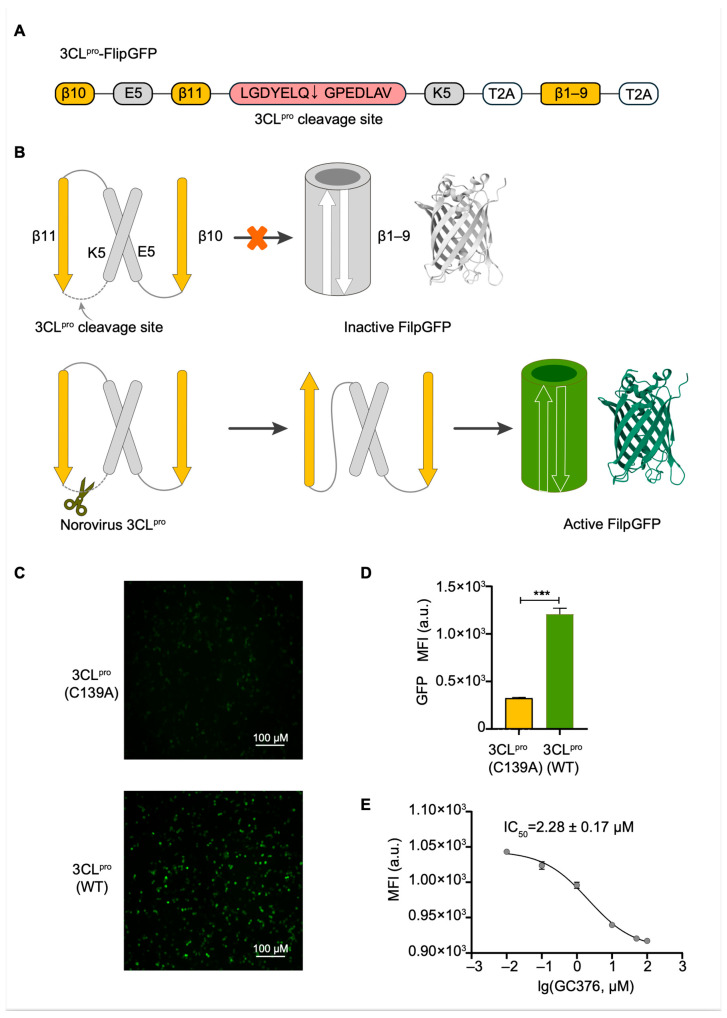
Development of the FlipGFP-3CL^pro^ assay. (**A**) Sequence of the 3CL^pro^-FlipGFP construct. T2A, a short peptide sequence derived from viral origins, possesses a unique self-cleavage property. K5, a coiled-coil forming peptide, pairs with its complementary partner peptide E5. They dimerize and induce the alignment of strands into a parallel conformation. (**B**) Schematic representation of the FlipGFP assay principle. Cleavage of the linker in the β10–11 fragment restores GFP fluorescence. (**C**) Fluorescence images of HEK293T cells expressing FlipGFP and mutant 3CL^pro^ (C139A) or wild-type 3CL^pro^. (**D**) Quantification of fluorescence intensity using a high-content screening system. (**E**) Dose–response curve of GC376 inhibition of 3CL^pro^, determined using the FlipGFP assay. Error bars represent mean ± SD (n = 3). Statistical significance was assessed using an unpaired Welch’s *t*-test (two-sided): *** *p* ≤ 0.001.

The FlipGFP assay offers a real-time, cell-based readout of protease activity, enabling direct observation of inhibitor effects in a physiological context [21]. In this assay, green fluorescent protein (GFP) was divided into 11 β-strands, with β1–9 forming a β-barrel structure and β10–11 separated from it. These segments were connected using a Norovirus 3CL^pro^-specific cleavage sequence, flanked by heterodimerizing coiled-coil domains E5 and K5 (Figure 1A) [21,22]. In the presence of Norovirus 3CL^pro^, the cleavage of its substrate sequence allows β10–11 to align antiparallel to the β1–9 barrel, restoring fluorescence intensity (Figure 1B). Conversely, the addition of a protease inhibitor prevents cleavage, reducing fluorescence to baseline levels. Given the high similarity of 3CL^pro^ proteins within the same genogroup (~90%) and moderate homology among genogroups (~50–70%), this system offers the potential to identify broad-spectrum inhibitors targeting multiple Norovirus genogroups. To optimize cleavage efficiency, we selected a reported 3CL^pro^ cleavage sequence [23] and inserted it within the GFP construct (Figure 1A). To validate this assay, we co-transfected of 3CL^pro^-FlipGFP and either wild-type (WT) 3CL^pro^ or its catalytically inactive mutant C139A [24,25] into HEK293T cells. Fluorescence imaging revealed robust GFP expression in the presence of 3CL^pro^(WT), whereas cells expressing the 3CL^pro^(C139A) mutant exhibited minimal fluorescence (Figure 1C). These results confirm that the increased fluorescence is dependent on the proteolytic activity of 3CL^pro^. A quantitative analysis using a high-content screening system further corroborated these findings, demonstrating a significant increase in fluorescence intensity in the presence of wild-type 3CL^pro^ compared to the control (Figure 1D).

To evaluate the system’s utility for inhibitor screening, we tested the known 3CL^pro^ inhibitor GC376 as a positive control [13]. HEK293T cells were co-transfected with 3CL^pro^-FlipGFP and 3CL^pro^ constructs, followed by the addition of GC376 six hours post-transfection. Fluorescence intensity was measured 24 h post-transfection, and the IC_50_ value was determined [26]. The calculated IC_50_ of 2.28 ± 0.17 μM for GC376 aligns with previously reported data [13] (Figure 1E), confirming the assay’s reliability for identifying 3CL^pro^ inhibitors. The assay’s sensitivity, real-time readout, and compatibility with high-throughput screening make it a valuable addition to the toolkit for Norovirus drug discovery.

### 3.2. Screening of 3CL^pro^ Protease Inhibitors by Virtual Screening

**Figure 2:**
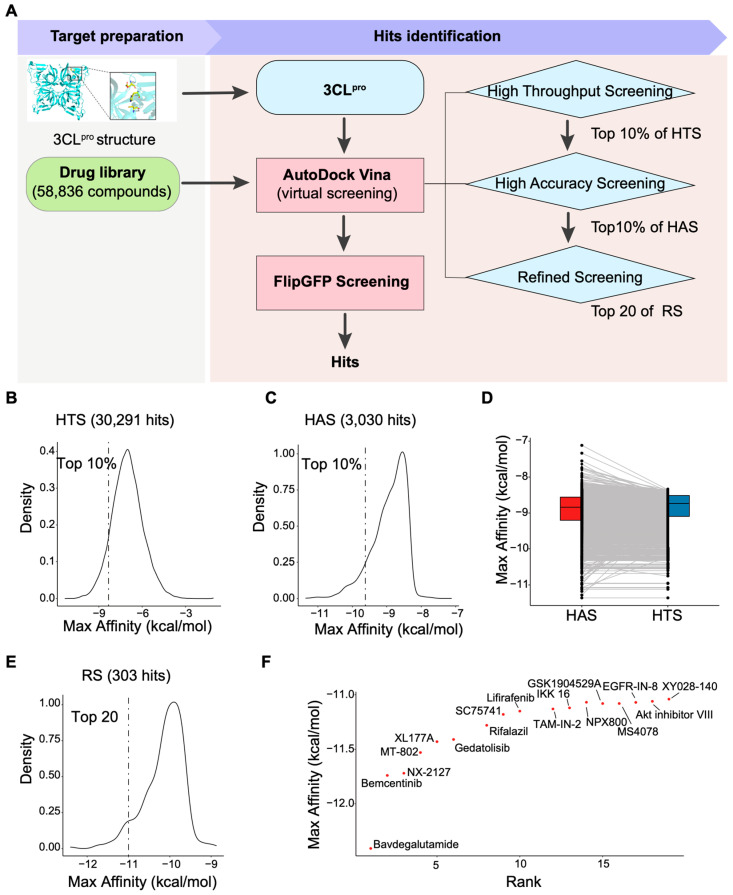
Virtual screening of potential 3CL^pro^ protease inhibitors. (**A**) Workflow for identifying 3CL^pro^ inhibitors. Structure of 3CL^pro^ (PDB ID: 8U1V). The catalytic triad (His30, Glu54, Cys139) is highlighted within the dashed box. (**B**) Line chart of Max Affinity scores from high-throughput screening, and high-accuracy screening (**C**). (**D**) Pairwise plot of Max Affinity scores from HTS and HAS. (**E**) Line chart of Max Affinity scores from refined screening. (**F**) Ranking of 17 candidate compounds based on Max Affinity scores.

To identify the candidate inhibitors of 3CL^pro^ protease, we employed computer-based virtual screening to identify small-molecule compounds that interact strongly with 3CL^pro^ [27]. The crystal structure of 3CL^pro^ (PDB: 8U1V) was retrieved from the Protein Data Bank (Figure 2A and Figure S1) and used as the docking target. To ensure comprehensive coverage of chemical space and enhance biological relevance, five compound libraries were selected for virtual screening: (1) the Golden Scaffold Library (5000 compounds) to ensure scaffold diversity; (2) the Selleck Compound Library (20,550 compounds) to include bioactive molecules from the ChEMBL database; (3) the Inhibitor Library (7315 compounds) enriched in known enzyme inhibitors; (4) the Immunology and Inflammation Library (14,036 compounds) to cover immune-related chemical space; (5) the FDA-approved Drug Library (11,935 compounds) to facilitate potential drug repurposing. This combined strategy balances structural diversity, target specificity, and clinical relevance. A combined compound library comprising 58,836 compounds (Table 1) was prepared, and OpenBabel was utilized to convert the compounds into the pdbqt format for docking [28]. The docking process was performed using AutoDock Vina, with a defined docking box set around the active site of 3CL^pro^ (Figure 2A).

To enhance the accuracy of the screening, we conducted a three-step virtual screening protocol with progressively refined parameters (exhaustiveness = 1, 8, 16). The first round, a high-throughput screening (HTS), aimed to rapidly identify compounds with potential binding affinity to 3CL^pro^. This initial screening yielded 30,291 compounds based on Max Affinity scores [29,30] (Figure 2B). In the second round, the top 10% of compounds from the HTS (3030 compounds) were subjected to more stringent docking parameters (high-accuracy screening, HAS) to improve binding stability and affinity (Figure 2C,D). Finally, the top 10% of compounds from the second round (303 compounds) were advanced to a refined screening to further optimize binding predictions (Figure 2E).

Binding affinities were categorized into four tiers: very good binding (≤−10 kcal/mol), good binding (−7 to −10 kcal/mol), moderate binding (−5 to −7 kcal/mol), and weak binding (>−5 kcal/mol) [30]. Among the 303 compounds screened in the final round, 166 exhibited very good binding affinity (≤−10 kcal/mol) (Table S1). Based on Max Affinity scores, the top 20 compounds were selected, and 17 were procured for experimental validation (Figure 2F).

### 3.3. Identifying Candidate Inhibitors Using a Cell-Based FlipGFP Assay

**Figure 3:**
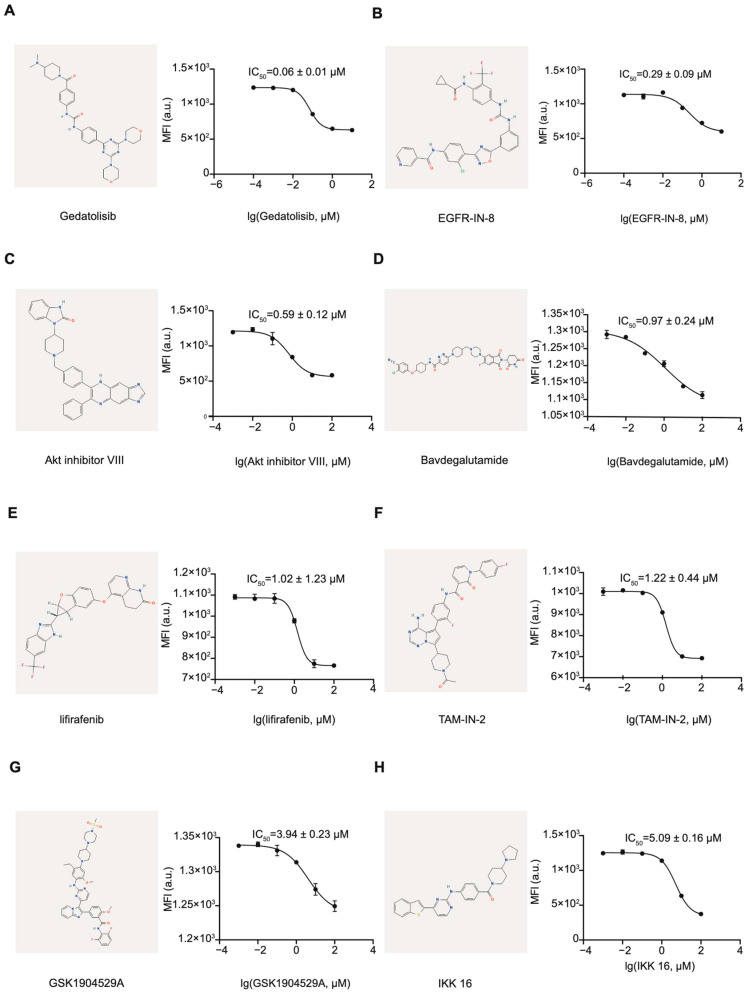
Structure and inhibition activity of hit compounds in the FlipGFP Assay. IC_50_ curves for (**A**) Gedatolisib, (**B**) EGFR-IN-8, (**C**) Akt inhibitor VIII, (**D**) Bavdegalutamide, (**E**) Lifirafenib, (**F**) TAM-IN-2, (**G**) GSK1904529A, and (**H**) IKK 16.

To evaluate the inhibitory effects of the 17 candidate compounds identified through virtual screening, we employed the established FlipGFP assay in HEK293T cells. The candidate compounds were added six hours post-transfection, and fluorescence intensity was quantified 24 h post-transfection using a high-content cell imaging system. Dose–response curves were generated, and IC_50_ values were calculated from triplicate experiments. Among the tested compounds, eight demonstrated significant inhibitory activity against 3CL^pro^: Gedatolisib (IC_50_ = 0.06 ± 0.01 μM), EGFR-IN-8 (IC_50_ = 0.29 ± 0.09 μM), Akt inhibitor VIII (IC_50_ = 0.59 ± 0.12 μM), Bavdegalutamide (IC_50_ = 0.97 ± 0.24 μM), Lifirafenib (IC_50_ = 1.02 ± 1.23 μM), TAM-IN-2 (IC_50_ = 1.22 ± 0.44 μM), GSK1904529A (IC_50_ = 3.94 ± 0.23 μM), and IKK 16 (IC_50_ = 5.09 ± 0.16 μM) (Figure 3 and Figure S2). Notably, Gedatolisib, which docking affinity with a Max Affinity score of −11.41 kcal/mol (Table 2), exhibited the most potent inhibitory effect (Figure 3A). These results validate the utility of the FlipGFP assay for identifying and characterizing 3CL^pro^ inhibitors, highlighting Gedatolisib as a candidate for further development. The combination of computational screening and cell-based validation provides a robust framework for discovering novel antiviral agents targeting Norovirus.

To further support the inhibitory effect on 3CL^pro^, we performed a Western blot assay to detect the cleavage of FlipGFP (Figure S3). Unexpectedly, two protein bands (~35 and ~25 kDa) were observed in the control group co-transfected with the catalytically inactive 3CL^pro^ mutant (C139A) and FlipGFP, suggesting the presence of multiple translation initiation sites in the FlipGFP construct. Consistently with this, transfection with wild-type 3CL^pro^ significantly reduced the intensity of both bands, indicating protease-mediated cleavage.

To determine whether the obtained compounds directly inhibit 3CL^pro^ activity, we further conducted Western blot experiments using representative compounds Gedatolisib and EGFR-IN-8 (Figure S3). Compared with the control group transfected with the inactive C139A mutant, cells treated with low concentrations of these inhibitors still exhibited substantial cleavage of FlipGFP. However, at higher inhibitor concentrations, proteolytic cleavage was markedly reduced, and the band pattern resembled that of the C139A control group. These results suggest that the compounds can directly inhibit 3CL^pro^ enzymatic activity in a dose-dependent manner.

### 3.4. Crystal Structures of Norovirus 3CL^pro^ in Complex with Hit Inhibitors

**Figure 4:**
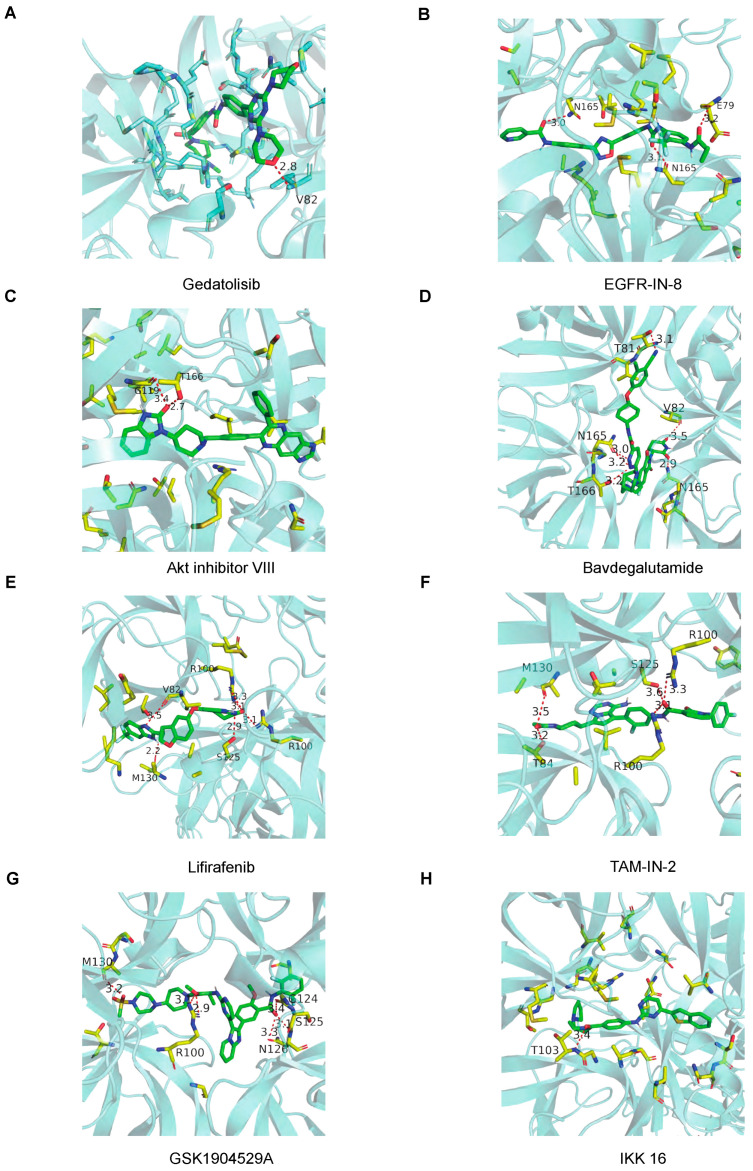
Structural interactions of hit compounds with 3CL^pro^. Three-dimensional visualization of Norovirus 3CL^pro^ in complex with (**A**) Gedatolisib, (**B**) EGFR-IN-8, (**C**) Akt inhibitor VIII, (**D**) Bavdegalutamide, (**E**) Lifirafenib, (**F**) TAM-IN-2, (**G**) GSK1904529A, and (**H**) IKK 16, highlighting key interacting residues.

To elucidate the inhibitory mechanisms of the eight candidate compounds, we analyzed their co-crystal structures with 3CL^pro^, focusing on the interactions between the compounds and key protease residues. All eight compounds were found to occupy the central cavity of the protease, forming hydrogen bonds and van der Waals interactions with multiple residues, which appear to underpin their inhibitory effects (Figure 4A–H). Notably, the identification of shared interacting residues across different compounds suggests their functional importance in maintaining protease activity. For example, Gedatolisib, Bavdegalutamide, and Lifirafenib—compounds with particularly high inhibitory activity—all formed hydrogen bonds with the Val82 residue, with Max Affinity scores of −11.41, −12.41, and −11.15 kcal/mol, respectively. This finding highlights Val82 as a critical residue beyond the catalytic triad (His30, Cys139, Glu54) that may play a pivotal role in inhibitor binding. Similarly, EGFR-IN-8 and Bavdegalutamide both formed hydrogen bonds with Asn165, while Lifirafenib, TAM-IN-2, and GSK1904529A shared interactions with Ser125, Met130, and Arg100. These results suggest that residues such as Asn165, Arg100, Ser125, and Met130 are also key targets for inhibitor design. Collectively, these structural insights provide a molecular basis for the observed inhibitory activities and highlight critical residues that could be leveraged in the rational design of next-generation anti-Norovirus drugs.

### 3.5. Molecular Dynamics Simulation of Hit Compound–3CL^pro^ Complexes

**Figure 5:**
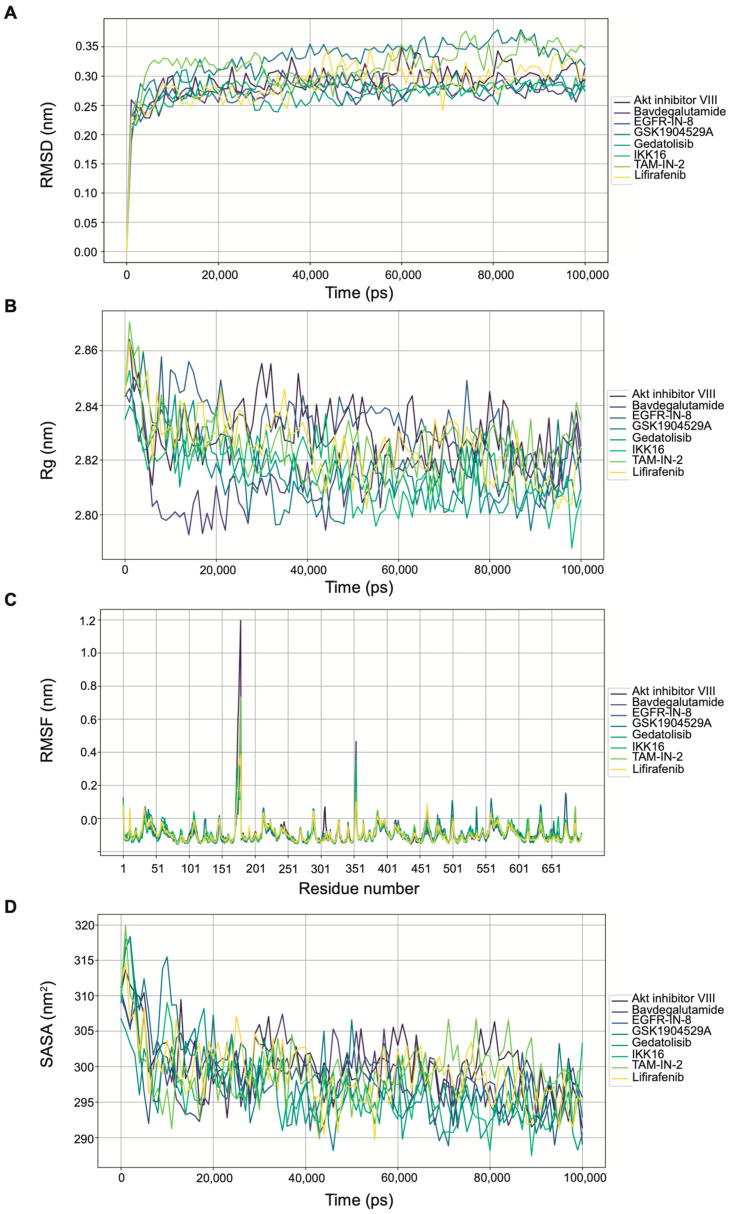
Molecular dynamics simulation of hit compound–3CL^pro^ complexes. (**A**) RMSD plots showing stable trajectories for all eight complexes over 100 ns. (**B**) Rg plots indicating compactness of the complexes. (**C**) RMSF plots highlighting regions of flexibility, particularly residues 165–180 and 345–355. (**D**) SASA plots demonstrating stability of the complexes during simulation.

To further investigate the structural stability of the complexes formed between the hit compounds and 3CL^pro^, we performed molecular dynamics (MD) simulations over a 100 ns timescale. The root mean square deviation (RMSD) analysis revealed that all eight complexes reached a stable equilibrium during the simulation, with RMSD values fluctuating within a narrow range (Figure 5A). Notably, the complexes of Gedatolisib, EGFR-IN-8, and Bavdegalutamide exhibited particularly stable RMSD values, ranging between 0.25 and 0.30 nm, indicating sustained binding to the protease and consistent inhibition of its activity.

To assess the folding and compactness of the complexes, we analyzed the radius of gyration (Rg). The Rg values for all complexes remained stable throughout the simulation, with fluctuations not exceeding 0.05 nm. The Gedatolisib–3CL^pro^ complex demonstrated exceptional stability, with Rg fluctuations of less than 0.02 nm between 20 and 100 ns (Figure 5B), suggesting a tight and stable binding interaction that aligns with its potent inhibitory activity observed in cell-based assays.

A root mean square fluctuation (RMSF) analysis was conducted to evaluate the flexibility of amino acid regions within the complexes. Large fluctuations were primarily observed in residues 165–180 and 345–355, regions likely associated with loop flexibility. Among the complexes, Akt inhibitor VIII exhibited the highest RMSF values in these regions (Figure 5C), indicating greater flexibility and potential instability compared to the other complexes.

Finally, a solvent-accessible surface area (SASA) analysis revealed minimal changes in the solvent-exposed surface area for all eight complexes, with small fluctuations observed throughout the simulation (Figure 5D). This suggests that the overall structural integrity and solvent accessibility of the complexes remained consistent, further supporting their stability.

In summary, MD simulations provided critical insights into the structural stability and binding dynamics of the hit compound–3CL^pro^ complexes. The results underscore the potential of these compounds, particularly Gedatolisib, as promising candidates for anti-Norovirus drug development, with stable and sustained interactions that effectively inhibit protease activity.

## 4. Discussion

Proteases represent a critical class of enzymes involved in a wide array of biological processes across viruses, bacteria, and eukaryotes. The dysregulation of protease activity is implicated in numerous diseases, making proteases—both host and viral—attractive targets for therapeutic intervention [1]. Viral proteases, in particular, play essential roles in viral replication and have been successfully targeted in the development of antiviral therapies, as exemplified by inhibitors of human immunodeficiency virus (HIV) [31] and hepatitis C virus (HCV) proteases [32]. Norovirus 3CL^pro^, a cysteine protease with chymotrypsin-like folds, consists of an N-terminal antiparallel β-sheet domain and a C-terminal β-barrel domain, with the catalytic triad (Cys139, His30, and Glu54) located in the cleft between these domains (Figure 2A and Figure S1). The substrate specificity of 3CL^pro^ is well conserved across Norovirus genogroups (Figure 1C), underscoring its potential as a broad-spectrum antiviral target.

In this study, we employed a combination of molecular docking, molecular dynamics (MD) simulations, and a cell-based FlipGFP assay to identify and validate small-molecule inhibitors of Norovirus 3CL^pro^. Notably, many of the identified compounds, including those with the strongest inhibitory activity, have previously been investigated for their anti-tumor, anti-inflammatory, or antibacterial properties (Table S1). For instance, Gedatolisib and Akt inhibitor VIII, both inhibitors of the PI3K-AKT-mTOR pathway [33,34], exhibit potent anti-tumor activity. Similarly, Lifirafenib and EGFR-IN-8, which target the epidermal growth factor receptor (EGFR) [35,36], have demonstrated efficacy in inhibiting tumor growth and metastasis. Bavdegalutamide, a proteolysis-targeting chimera (PROTAC) degrader of the androgen receptor (AR), shows promise in prostate cancer treatment [37], while TAM-IN-2, a TAM receptor inhibitor, effectively blocks Gas6-induced AXL activation and suppresses lung cancer progression. Additionally, IKK16, a selective inhibitor of IκB kinase (IKK), modulates inflammatory responses [38]. Our current findings only provide initial evidence that these compounds can inhibit protease activity. Further validation through live virus assays and in vitro antiviral tests is necessary to confirm their ability to suppress viral replication. In summary, these findings suggest that repurposing existing anti-tumor and anti-inflammatory drugs may offer a promising strategy for Norovirus drug development, though further structural optimization and clinical validation are required.

## 5. Conclusions

In summary, this study integrates a cell-based FlipGFP assay with computational virtual screening to identify and validate small-molecule inhibitors of Norovirus 3CL^pro^. The identified compounds, many of which have established roles in oncology and inflammation, provide a foundation for the design and repurposing of therapeutics targeting Norovirus. Our findings highlight the potential of leveraging existing drug libraries to accelerate the discovery of antiviral agents and offer new insights into the development of broad-spectrum Norovirus treatments.

## Figures and Tables

**Table 1:** Composition of the compound library for virtual screening.

Library	Number	Description
Golden Scaffold Library	5000	Representative drug-like compounds selected from the ChemDiv core library, covering 5000 skeletal structures
Selleck Compound Library	20,550	Representative bioactive molecules from the ChEMBL database
Inhibitor Library	7315	Representative known inhibitors
Immunology or Inflammation Compound Library	14,036	Multiple compounds related to immune inflammation from MCE Company
Approved FDA Drugs Database	11,935	Preclinical, clinical, and FDA-approved compounds
Total number of compounds	58,836	

**Table 2:** Physicochemical properties, Max Affinity scores, and hydrogen-bonded residues of 3CL^pro^–hit complexes.

PubChem ID	Compound Name	Max Affinity (kcal/mol)	H-Bonded Residues
2222112-77-6	Bavdegalutamide	−12.41	Thr81, Val82, Asn165 and Thr166
44516953	Gedatolisib	−11.41	Val82
1446090-79-4	Lifirafenib	−11.15	Val82, Arg100, Ser125 and Met130
2135642-56-5	TAM-IN-2	−11.13	Thr84, Arg100, Ser125 and Met130
9549298	IKK 16	−11.12	Thr103
1089283-49-7	GSK1904529A	−11.08	Arg100, Gly124, Ser125, Asn126 and Met130
139035057	EGFR-IN-8	−11.07	Glu79 and Asn165
135398501	Akt inhibitor VIII	−11.06	Gly119 and Thr166

## Data Availability

The data are contained within the article.

## Supplementary Materials

The following supporting information can be downloaded at: https://www.mdpi.com/article/10.3390/v17060814/s1, Table S1: Physicochemical properties, Max Affinity scores, and targets of 303 hits from refined screening. Figure S1: Structural representation of the norovirus main protease (3CLpro), highlighting key catalytic and substrate-binding residues. Figure S2: High-resolution structural representations of the eight small-molecule inhibitors screened against norovirus 3CL^pro^. Figure S3: Western blot analysis of FlipGFP cleavage to assess the enzymatic activity of 3CL^pro^ and its inhibition by selected compounds.

## Abbreviations

The following abbreviations are used in this manuscript:

3CL^pro^	3C-like cysteine protease
GFP	Green fluorescent protein
WT	Wild-type
HTS	High-throughput screening
HAS	High-accuracy screening
MD	Molecular dynamics
RMSD	Root mean square deviation
Rg	Radius of gyration
RMSF	Root mean square fluctuation
HIV	Human immunodeficiency virus
HCV	Hepatitis C virus
PROTAC	Proteolysis-targeting chimera
EGFR	Epidermal growth factor receptor
AR	Androgen receptor
IKK	IκB kinase
SASA	Solvent-accessible surface area
