# Supplementary figures and images for: Discovery of Small-Molecule Inhibitors Against Norovirus 3CLpro Using Structure-Based Virtual Screening and FlipGFP Assay
Source: Viruses. 2025 Jun 4;17(6):814. doi: 10.3390/v17060814 (PMC12197363; doi:10.3390/v17060814)

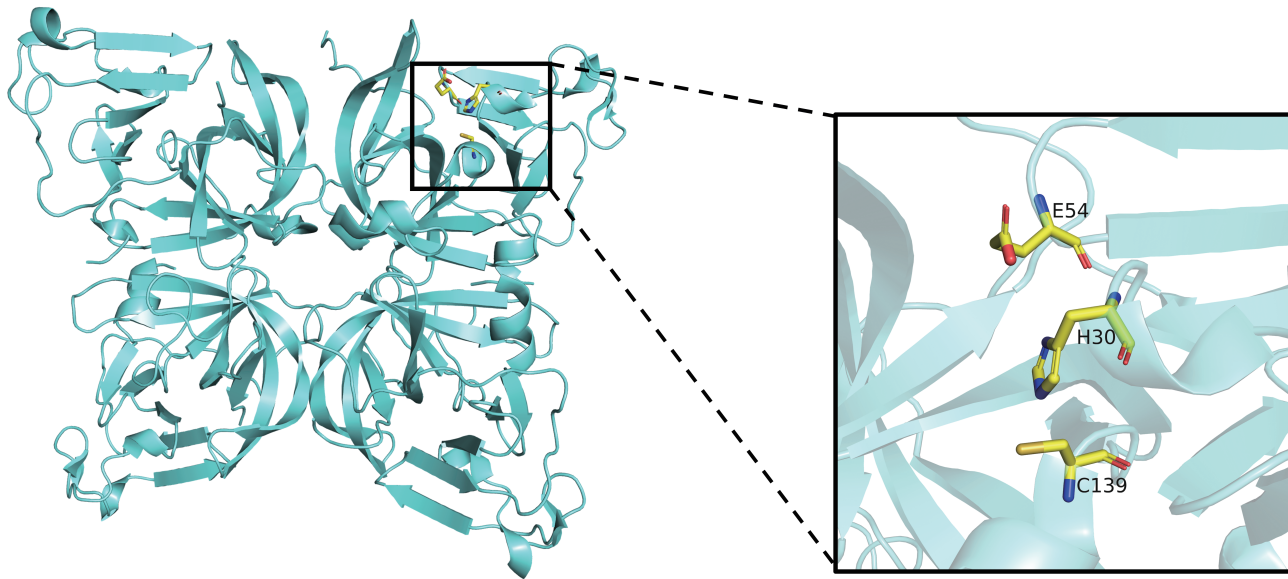

Supplement: Supplementary file 1 [file viruses-17-00814-s001.zip › Figure S1.pdf]

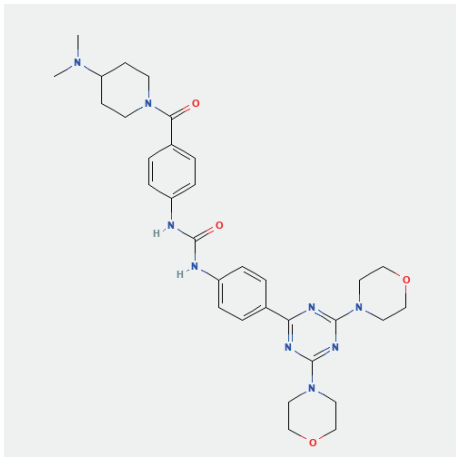

Gedatolisib

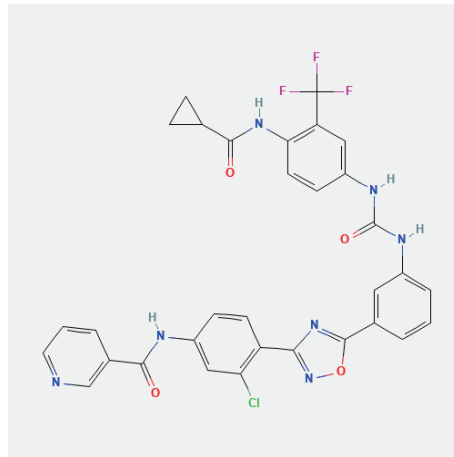

EGFR-IN-8

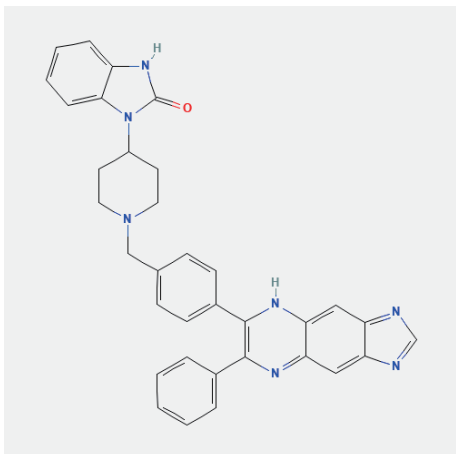

Akt inhibitor VIII

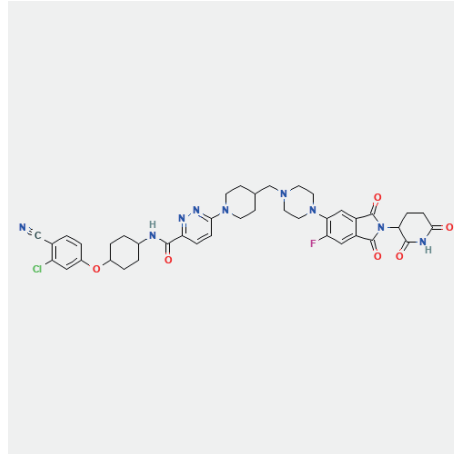

Bavdegalutamide

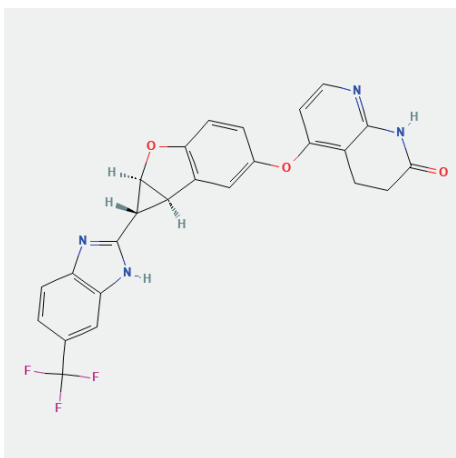

lifirafenib

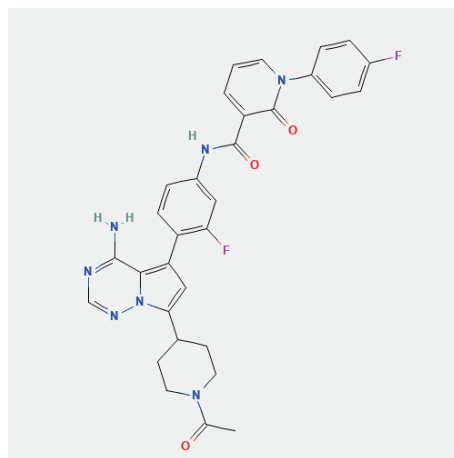

TAM-IN-2

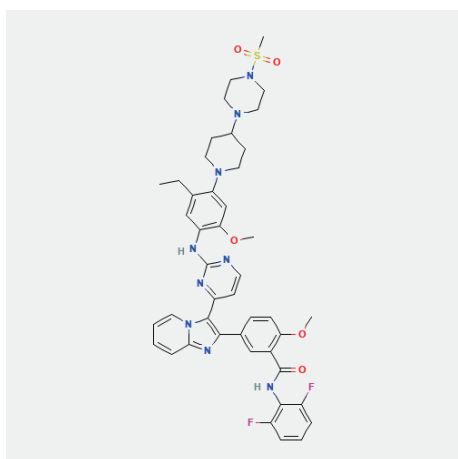

GSK1904529A

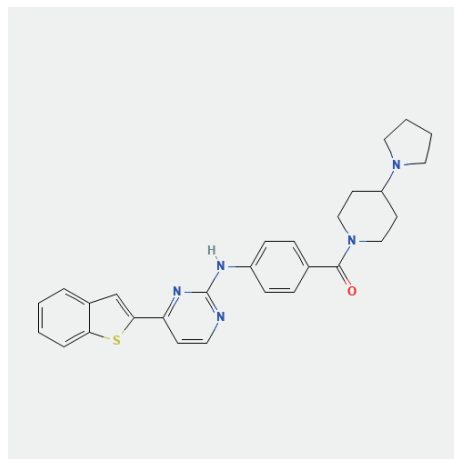

IKK 16

Supplement: Supplementary file 1 [file viruses-17-00814-s001.zip › Figure S2.pdf]

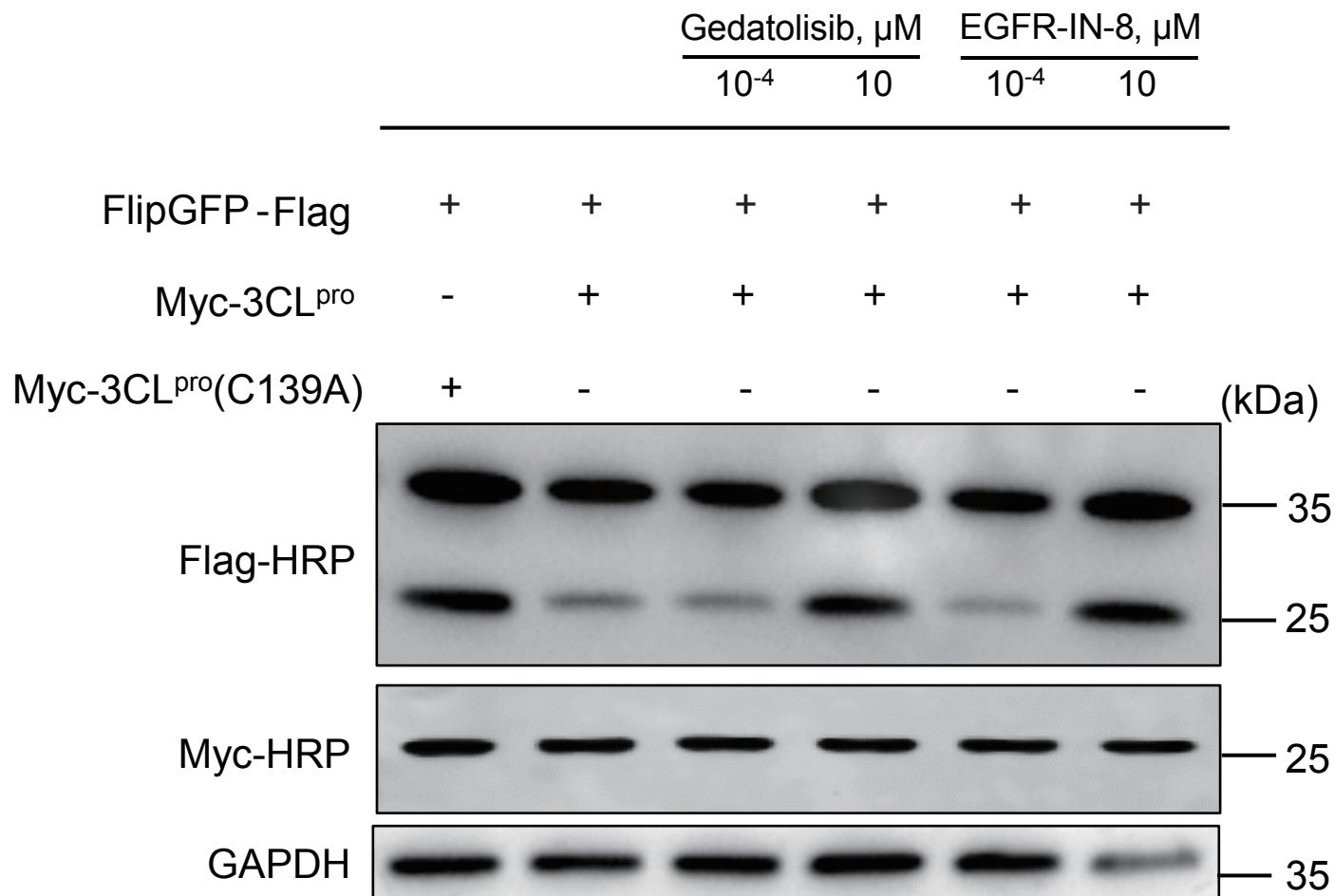

Supplement: Supplementary file 1 [file viruses-17-00814-s001.zip › Figure S3.pdf]
